# Brain Structure as a Correlate of Odor Identification and Cognition in Type 2 Diabetes

**DOI:** 10.3389/fnhum.2022.773309

**Published:** 2022-02-14

**Authors:** Mimi Chen, Jie Wang, Shanlei Zhou, Cun Zhang, Datong Deng, Fujun Liu, Wei Luo, Jiajia Zhu, Yongqiang Yu

**Affiliations:** ^1^Department of Radiology, The First Affiliated Hospital of Anhui Medical University, Hefei, China; ^2^Department of Endocrinology, The First Affiliated Hospital of Anhui Medical University, Hefei, China; ^3^Department of Radiology, Chaohu Hospital of Anhui Medical University, Chaohu, China

**Keywords:** type 2 diabetes, magnetic resonance imaging, cortical thickness, cognitive function, olfactory function

## Abstract

**Background**: It has been reported that type 2 diabetes (T2DM) is associated with olfactory identification (OI) impairments and cognitive decline. However, the relationship between OI impairments and cognitive decline is largely unknown in T2DM patients.

**Methods**: Sixty-eight T2DM patients and 68 healthy controls underwent 3D-T1 MRI scans, olfactory and cognitive assessments. The cortical thickness of olfaction-related brain regions, olfactory and cognitive scores were compared between groups. Correlation analyses were carried out among cognition, olfaction, and cortical thickness of olfaction-related brain regions.

**Results**: First, the cognitive and olfactory test scores of T2DM patients were lower than healthy subjects. Second, higher olfactory scores were associated with increased cortical thickness in the left parahippocampal gyrus and bilateral insula in T2DM. Third, higher olfactory scores were associated with higher cognitive performance in T2DM. Fourth, some cognitive performances were related to cortical thickness in the left parahippocampal gyrus and left insula in T2DM.

**Conclusion**: These findings indicated that olfactory dysfunction may be useful for future applications that attempt to predict cognitive decline or develop tailored therapies in T2DM patients.

## Introduction

Diabetes is a chronic metabolic disorder, which is characterized by hyperglycemia. Long-term hyperglycemia can cause various complications such as kidney diseases, eye diseases, neuropathy, etc. Type 2 diabetes (T2DM) is the most common type of diabetes in adults (Sanchez-Brito et al., 2021). The global prevalence of T2DM continues to rise. It was estimated that 463 million people had diabetes in 2019, accounting for 9.3% of the global adult population (20–79 years old). This number is expected to increase to 578 million (10.2%) by 2030 and 700 million (10.9%) by 2045 (Saeedi et al., 2019). Previous studies have shown that chronic hyperglycemia can cause cognitive dysfunction (Cao et al., 2020). Cognitive impairmentincludes loss of memory, visual-spatial ability, language ability, abstract thinking, executive function, etc. (Yu et al., 2020). Neuropsychological tests demonstrated that the rate of cognitive impairment of T2DM patients has exceeded that of age-related cognitive decline, especially in terms of learning and memory, executive function, and attention (Weinstein et al., 2018). The incidence of mild cognitive impairment (MCI) among T2DM patients has increased by 50%–60%. MCI is the prodromal stage of Alzheimer’s disease (AD), and diabetes can accelerate the transition from MCI to AD (Li et al., 2020; Liu et al., 2021). Therefore, early diagnosis and treatment intervention for T2DM patients with MCI may reduce the occurrence and deterioration of cognitive impairment (Li et al., 2020).

Recently, magnetic resonance imaging (MRI) has provided a non-invasive examination to detect brain structure changes, which is conducive to the early diagnosis and treatment of diseases. A growing body of literature has indicated that brain structural changes in diabetes patients contribute to their cognitive decline (Bruehl et al., 2009). By using structural MRI, brain atrophy was found present in dementia-free middle-aged adults with T2DM. Regional brain atrophy appears to be developing even in the subgroup that shows no clinical evidence of microvascular disturbances (Fang et al., 2018). There was evidence that gray matter atrophy associated with T2DM is widely and bilaterally distributed in hippocampi, temporal, frontal, cingulate cortices, and subcortical nuclei. It appears to be the primary driver of cognitive dysfunction in people with T2DM (Moran et al., 2013). An alternative strategy to quantify gray matter morphometric abnormalities involves the use of surface-based methods that can measure cortical thickness (Messina et al., 2013). Li et al. (2018) found changes in cortical thickness of multiple brain regions in T2DM patients, and the cortical thickness reduction of the right pars opercularis may play an important role in the pathophysiological mechanism of cognitive impairment and serve as a biomarker. Surface-based cortical thickness is an accurate tool to detect focal cortical atrophy (Takayanagi et al., 2020). Furthermore, previous studies have confirmed that the surface-based method is less susceptible to partial volume effects than the voxel-based morphometry (VBM) method (Clarkson et al., 2011). Therefore, we choose cortical thickness as our main measurement index.

Smell is an external sensation produced by odor molecules to stimulate the body. Almost all living things can recognize odors. As an intuitive human sense, smell plays an irreplaceable role in identifying harmful gases, choosing food, promoting appetite, affecting emotions, and warning of danger. It also participates in immune regulation and cognitive functions (Rolls, 2019). Studies have shown that compared with control subjects with normal cognition, AD patients have olfactory dysfunction, which was significantly related to cognitive impairment severity (Wang et al., 2010; Wu et al., 2019). Furthermore, previous studies have proved that diabetic patients have lower olfactory scores, and olfactory dysfunction occurs before cognitive decline (Zhang et al., 2018, 2019).

There are many olfactory test methods, such as the olfactory testing in Parkinsonism, the Sniffin’ sticks test, the University of Pennsylvania smell identification test (UPSIT), and the Connecticut Chemosensory Clinical Research Center olfactory test (CCCRC). The olfactory test results may be affected by cultural background and region. Therefore, in this study, The Chinese smell identification test (CSIT; Feng et al., 2019) was used to detect olfactory performance. We examined the differences of olfactory performance between T2DM patients and healthy subjects. What’s more, correlation analyses were performed among olfactory scores, the cortical thickness of olfaction-related brain regions and cognition scores to investigated the relationships between olfactory performance, brain structure, and cognitive function in T2DM patients.

## Materials and Methods

### Participants

This study included a total of 136 participants, including 68 T2DM patients and 68 healthy controls (HC), who were matched by age, sex, and education. T2DM patients are all inpatients from the department of endocrinology in the First Affiliated Hospital of Anhui Medical University. The HC were recruited from communities near the hospital by advertisements or from family members of the T2DM patients. All participants were right-handed, aged between 30 and 65 years old, with more than 6 years of education. The diagnosis of patients with T2DM was based on the standards of the American Diabetes Association and the duration of diabetes were more than 1 year (American Diabetes Association, 2021). All the HC participants had normal glucose tolerance. The cognitive function of all participants was assessed by Montreal Cognitive Assessment (MoCA; scores ≥19; Nasreddine et al., 2005). Exclusion criteria for all participants were: (1) history of severe somatic disease such as thyroid dysfunction, cardiovascular disease, severe liver and kidney disease, etc.; (2) neurological or psychiatric disorders; (3) nasal pathologies affecting olfactory function such as acute or chronic sinusitis, allergic rhinitis, nasal polyposis, deviated nasal septum, and a history of nasal trauma or surgery; (4) cerebrovascular disease such as cerebral hemorrhage, cerebral infarction, tumor, and history of trauma or surgery; (5) inability to complete cognitive and olfactory tests; and (6) MRI contraindications such as claustrophobia, metal implants in the body, etc. The Research Ethics Committee of the First Affiliated Hospital of Anhui Medical University approved this study. Written informed consent was obtained from all participants before registration.

### Cognitive Assessments

The cognitive assessments include the Auditory Verbal Learning Test (AVLT), the Mini-mental State Examination (MMSE), the Montreal Cognitive Assessment (MoCA; Nasreddine et al., 2005), the Trail Making Test-A (TMT-A), the Symbol Digital Modalities test (SDMT), the Digit Span Test (forward and backward), and the Animal Fluency Test (VFT). These tests cover multiple aspects such as memory, executive/attention function, visual-spatial perception, etc. All tests were performed in a fixed sequence by a well-trained neuropsychologist in a quiet environment and the subjects were relaxed and conscious.

### Olfactory Tests

The CSIT, designed by the Institute of Psychology, Chinese Academy of Sciences, can evaluate the performance of olfactory identification (OI; Feng et al., 2019). This test consists of two parts. The first part is a self-rating scale such as smoking history, drinking history, disease history, medication history, and self-assessment of smell. The second part is the 40 sniffing stick tests, similar to the UPSIT (Doty et al., 1984), but more suitable for people with a Chinese cultural background. Odorants of the CSIT were presented in felt-tip pens (Hummel et al., 1997), each filled with 1 ml of liquid. All odors are frequently touched in daily life, such as the odors of various fruits, nuts, spices, etc. The test was carried out in a quiet, ventilated, odorless, and dry environment. The instructors first removed the sniffing stick cap, placed the pen tip 1–2 cm in front of the subject’s nose, and swiped it slowly at a constant speed for 5 s. Then the subject needs to answer a multiple choice question with four options. The CSIT-OI total score is the number of tests with correct choices.

### MRI Data Acquisition

Structural MRI images were acquired on a 3.0-Tesla MR system (Discovery MR750w, General Electric, Milwaukee, WI, United States) with a 24-channel head coil. The brain volume (BRAVO) sequence was used with the following parameters to obtain high-resolution 3D T1-weighted structural images: repetition time (TR) = 8.5 ms; echo time (TE) = 3.2 ms, inversion time (TI) = 450 ms; flip angle (FA) = 12°, field of view (FOV) = 256 mm × 256 mm, matrix = 256 × 256; slice thickness = 1 mm, no gap; 188 sagittal slices; and acquisition time = 296 s. All subjects kept still and relaxed during the scans. No participants were excluded due to imaging artifacts on visual inspection.

### Image Processing and Surface-Based Morphometry Analyses

Surface-based morphometry (SBM) analysis was performed using the CAT12 toolbox^1^ implemented in the Statistical parametric mapping software (SPM12^2^). FreeSurfer’s standard automatic reconstruction algorithm was used to reconstruct the cortical surface, including the normalization of tissue intensity unevenness, the removal of non-brain tissues such as skin and skull, the conversion to Talairach-like space, and the segmentation of gray/white matter tissue. Cortical thickness measurements are obtained by calculating the shortest distance from the gray matter boundary to the white matter boundary (Desikan et al., 2006). Each image was resampled onto the average subject (average) and smoothed with a 15-mm, full-width, half maximum (FWHM) Gaussian kernel. We visually inspected the original structure image and segmentation quality of all participants. Cortical regions were segmented using the Desikan-Killiany atlas. We defined the bilateral entorhinal cortex, the lateral orbitofrontal cortex, the medial orbitofrontal cortex, parahippocampus, and insula as olfaction-related regions according to a previous study regarding olfactory dysfunction in T2DM and extracted the cortical thickness of these brain regions for the further region of interest (ROI)-level analyses (Zhang et al., 2019).

### Statistical Analysis

Demographic variables, cognitive test scores, olfactory test scores, and average cortical thickness of each ROI were compared between the T2DM and HC groups using two-sample *t*-tests. The gender difference was tested by using the Chi-square test between groups. Pearson correlation analyses were used to examine the associations among CSIT-OI scores, cortical thickness of the ROI, cognitive scores in T2DM and HC separately. All correlation analysis was corrected for multiple comparisons using the false discovery rate (FDR) method. These statistical analyses were performed by using the SPSS 21.0 software package.

## Results

### Demographic, Cognitive, Olfactory, and Olfaction-Related Regions’ Characteristics

**Figure 1:**
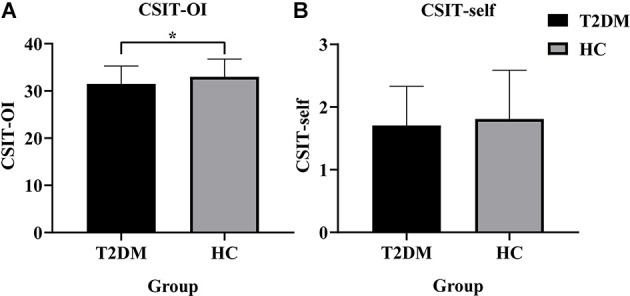
Histogram of CSIT scores for all participants in this study. **(A)** The CSIT-OI scores of patients with T2DM were lower than HC (*P < 0.05). **(B)** There was no difference in the CSIT-self scores of the two groups (*P* > 0.05). T2DM, Type 2 Diabetes Mellitus; HC, Healthy Controls; CSIT, Chinese Smell Identification Test; OI, olfactory identification.

Demographic, cognitive, and olfactory results of the sample are listed in Table 1. Two groups were matched for age (two-sample *t*-test, *t* = 1.473, *P* = 0.143), sex (Chi-square test, *χ^2^* = 0.369, *P* = 0.713) and education (two-sample *t*-test, *t* = −0.655, *P* = 0.514). Compared with HC subjects, T2DM subjects had significantly lower cognitive scores in the AVLT (delay; *t* = −2.274, *P* = 0.025), MoCA (*t* = -3.336, *P* = 0.001), SDMT (*t* = −3.406, *P* = 0.001), DST-forward (*t* = −2.395, *P* = 0.018), DST-backward (*t* = −2.201, *P* = 0.029) and VFT (*t* = −2.887, *P* = 0.005). The T2DM group exhibited significant lower scores in CSIT-OI (*t* = −2.360, *P* = 0.020), but not in CSIT-self (*t* = −0.851, *P* = 0.396; Figure 1). There were no significantly differences in the cortical thickness of olfaction-related regions between T2DM and healthy control groups.

**Table 1:** Demographic and clinical variables, cognitive assessment scores, and olfactory test scores.

	T2DM (*n* = 68)	HC (*n* = 68)	Statistics	*P*-value
Sex (men/women)^#^	48/20	46/22	*χ^2^* = 0.369	0.713
Age	47.71 ± 7.80	45.76 ± 7.57	*t* = 1.473	0.143
Education	13.59 ± 2.94	13.96 ± 3.58	*t* = −0.655	0.514
Duration of diabetes (years)	7.03 ± 6.04	-	-	-
**Cognitive assessment**				
AVLT (immediate)	8.93 ± 1.78	9.53 ± 1.91	*t* = −1.891	0.061
AVLT (delay)	9.10 ± 3.34	10.30 ± 2.74	*t* = −2.274	0.025*
AVLT (recognize)	13.82 ± 1.45	14.15 ± 1.36	*t* = −1.347	0.180
MMSE	28.87 ± 1.21	29.19 ± 0.83	*t* = −1.818	0.072
MoCA	25.96 ± 2.76	27.25 ± 1.62	*t* = −3.336	0.001*
TMT-A	38.51 ± 13.57	34.65 ± 13.40	*t* = 1.671	0.097
SDMT	47.43 ± 10.91	54.12 ± 11.92	*t* = −3.406	0.001*
DST-forward	7.85 ± 1.37	8.43 ± 1.42	*t* = −2.395	0.018*
DST-backward	5.37 ± 1.64	5.97 ± 1.56	*t* = −2.201	0.029*
VFT	38.01 ± 8.54	42.53 ± 9.66	*t* = −2.887	0.005*
**Olfactory test**				
CSIT-self^∧^	2 (1, 2)	2 (1, 2)	*t* = −0.851	0.396
CSIT-OI score	31.47 ± 3.80	33.00 ± 3.75	*t* = −2.360	0.020*

Data are mean ± standard, ^#^Chi-square test, ^∧^Kruskal–Wallis test. *P < 0.05 was considered significant. T2DM, Type 2 Diabetes Mellitus; HC, Healthy Controls; AVLT, Auditory Verbal Learning Test; MMSE, Mini-Mental State Examination; MoCA, Montreal Cognitive Assessment; TMT, Trail Making Test; SDMT, Symbol Digit Modalities Test; DST, Digit Span Test; VFT, Verbal Fluency Test; CSIT, Chinese Smell Identification Test.

### Correlations Between CSIT-OI Scores and Cortical Thickness of Olfaction-Related Regions

The correlations between CSIT-OI scores and cortical thickness are illustrated in Table 2. After FDR correction, we found significant positive correlations between CSIT-OI scores and cortical thickness in the left parahippocampal gyrus (*r* = 0.340, *P* = 0.030), left insula (*r* = 0.303, *P* = 0.040) and right insula (*r* = 0.328, *P* = 0.030) in T2DM groups. However, no correlations were found between CSIT-OI scores and cortical thickness of olfaction-related regions in HC groups.

**Table 2:** Correlation analysis of CSIT-OI scores and cortical thickness of the ROI.

	T2DM		HC	
	*r*	*P*	*r*	*P*
L-entorhinal cortex	0.030	0.808	0.287	0.180
R-entorhinal cortex	0.199	0.173	0.147	0.325
L-lateral orbitofrontal cortex	0.065	0.808	0.119	0.370
R-lateral orbitofrontal cortex	0.199	0.173	0.237	0.255
L-medial orbitofrontal cortex	−0.046	0.808	0.046	0.712
R-medial orbitofrontal cortex	0.037	0.808	0.161	0.325
L-parahippocampus	0.340*	0.030	0.138	0.325
R-parahippocampus	0.205	0.173	0.163	0.325
L-insula	0.303*	0.040	0.141	0.325
R-insula	0.328*	0.030	0.182	0.325

T2DM, Type 2 Diabetes Mellitus; HC, Healthy Controls; *r*, correlation coefficient (Pearson’s correlations); *P*, the *P*-value after false discovery rate (*FDR*) correction. *Correlation is significant at the 0.05 level. ROI, Region of Interesting; L, left; R, right.

### Correlations Between CSIT-OI Scores and Cognitive Test Scores

The correlations between CSIT-OI scores and cognitive test scores are illustrated in Table 3. After FDR correction, CSIT-OI scores exhibited significant positive correlations with MMSE (*r* = 0.257, *P* = 0.049), MoCA (*r* = 0.478, *P* < 0.001), SDMT (*r* = 0.323, *P* = 0.018), DST-forward (*r* = 0.376, *P* = 0.007), DST-backward (*r* = 0.264, *P* = 0.049), VFT (*r* = 0.265, *P* = 0.049), and negative correlation with TMT-A (*r* = −0.414, *P* < 0.001) in T2DM patients.

**Table 3:** Correlation analysis of CSIT-OI scores and cognitive test scores.

	T2DM		HC	
	*r*	*P*	*r*	*P*
AVLT (immediate)	0.235	0.068	0.213	0.104
AVLT (delay)	0.221	0.078	0.235	0.104
AVLT (recognize)	0.075	0.543	-0.100	0.422
MMSE	0.257*	0.049	0.138	0.289
MoCA	0.478**	<0.001	0.502*	0.001
TMT-A	-0.414**	<0.001	-0.248	0.102
SDMT	0.323*	0.018	0.304*	0.040
DST-forward	0.376*	0.007	0.328*	0.030
DST-backward	0.264*	0.049	0.212	0.104
VFT	0.265*	0.049	0.212	0.104

*r*, correlation coefficient (Pearson’s correlations); *P*, the *P-*value after false discovery rate (*FDR*) correction; *Correlation is significant at the 0.05 level; **Correlation is significant at the 0.001 level. T2DM, Type 2 Diabetes Mellitus; HC, Healthy Controls; AVLT, Auditory Verbal Learning Test; MMSE, Mini-Mental State Examination; MoCA, Montreal Cognitive Assessment; TMT, Trail Making Test; SDMT, Symbol Digit Modalities Test; DST, Digit Span Test; VFT, Verbal Fluency Test.

### Correlations Between Cognitive Test Scores and Cortical Thickness of Olfaction-Related Regions

**Figure 2:**
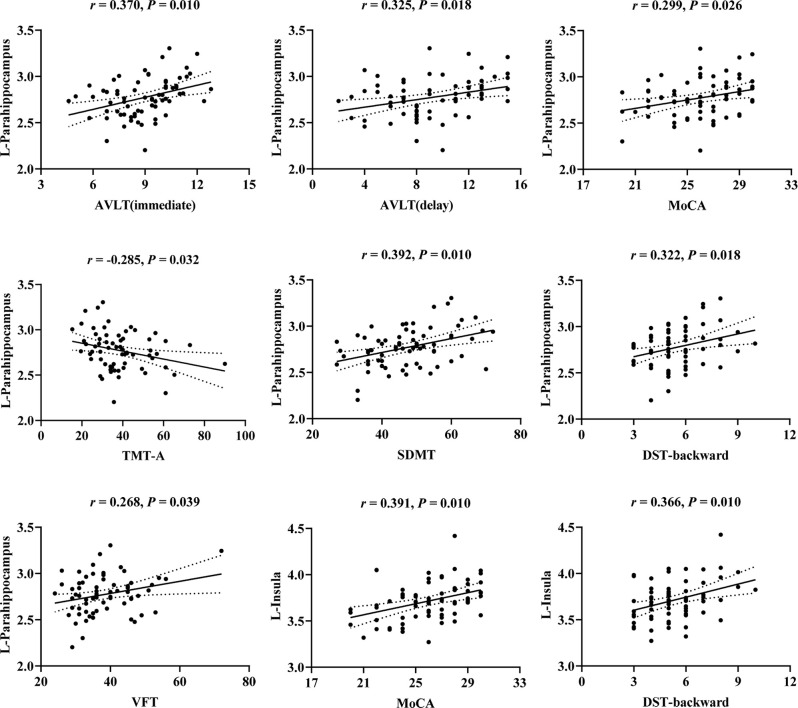
Scatter plots of the correlations between cognitive scores and cortical thickness of the left parahippocampus and left insula in T2DM. *r*, correlation coefficient (Pearson’s correlations); *P*, the *P*-value after false discovery rate (FDR) correction; *P* < 0.05 was considered significant. L, left; AVLT, Auditory Verbal Learning Test; MoCA, Montreal Cognitive Assessment; TMT, Trail Making Test; SDMT, Symbol Digit Modalities Test; DST, Digit Span Test; VFT, Verbal Fluency Test.

The correlations between cognitive scores and cortical thickness of olfaction-related regions in T2DM are illustrated in Figure 2. After FDR correction, cortical thickness in the left parahippocampal gyrus showed significant positive correlations with AVLT (immediate; *r* = 0.370, *P* = 0.010), AVLT (delay; *r* = 0.325, *P* = 0.018), MoCA (*r* = 0.299, *P* = 0.026), SDMT (*r* = 0.392, *P* = 0.010), DST-backward (*r* = 0.322, *P* = 0.018), VFT (*r* = 0.268, *P* = 0.039), and negative correlation with TMT-A (*r* = −0.285, *P* = 0.032). Cortical thickness in the left insula showed significant positive correlations with MoCA (*r* = 0.391, *P* = 0.010), DST-backward (*r* = 0.366, *P* = 0.010). However, there were no significant correlations between cognitive test scores and cortical thickness in the right insula (*P* > 0.05).

## Discussion

In this study, we examined the alterations of cognitive function, cortical thickness of olfaction-related regions, and olfactory test scores in T2DM, and the relationships among them in T2DM and healthy controls. Four main results were observed in this study. First, some cognitive and olfactory test scores were lower in T2DM patients. Second, higher CSIT-OI scores were associated with the increased cortical thickness of the left parahippocampal gyrus and bilateral insula in T2DM. Third, higher CSIT-OI scores were associated with higher cognitive scores in T2DM. Fourth, many cognitive testing scores were related to the cortical thickness of the left parahippocampal gyrus and left insula in T2DM.

As the only sensation directly connected to the external environment, smell plays an essential role in seeking food, predicting threats, and regulating interpersonal relationships and can affect individual emotional changes and participate in the development of various diseases (Nordin and Brämerson, 2008; Doty, 2017). Previous studies have found that T2DM patients have olfactory dysfunction through olfactory tests. Cross-sectional studies have shown that patients with diabetes have lower odor thresholds and discrimination recognition scores *via* using the University of Pennsylvania Smell Identification Test and “Sniffin” Sticks (Gouveri et al., 2014; Zaghloul et al., 2018). In this study, we used a new olfactory test method developed based on Chinese cultural background and found that T2DM patients showed worse olfactory recognition ability than healthy controls.

The cognitive dysfunction of diabetic patients has attracted a lot of attention in recent years (Livingston et al., 2017). There has been strong evidence shown that T2DM is an independent risk factor for cognitive dysfunction (Arnold et al., 2018). For instance, previous research has shown that diabetes could increase cognitive impairment risk, and the risk increases with age (Xue et al., 2019). It has been reported that the rate of diabetes-related cognitive decline is 50% faster than normal cognitive aging (Biessels and Despa, 2018). A meta-analysis showed that patients with T2DM showed a decrease in multiple cognitive subfields such as episodic memory and executive control (Sadanand et al., 2016). Consistent with previous studies, we found T2DM patients had lower cognitive test scores in AVLT, MoCA, SDMT, DST, and VFT.

The parahippocampal gyrus is related to cognitive processes and emotional editing, including visuospatial processing and episodic memory (Aguirre et al., 1996; Aminoff et al., 2013). Studies have found that the thinning of the cortex in the left parahippocampal gyrus was related to the decrease in olfactory function, and this phenomenon may reflect the first sign of olfactory impairment before pathological changes in the hippocampus, amygdala, and orbitofrontal cortex (Kubota et al., 2020). Liu et al. (2016) found that the connectivity of the left parahippocampal gyrus was reduced in AD patients, and the connectivity of the parahippocampal gyrus was related to the severity of the disease in MCI and AD subjects. A previous work revealed a significant negative correlation between the olfactory detection score with the volume of the left parahippocampal gyrus, and a positive correlation of the olfactory identification score with the Alzheimer’s disease assessment scale-cognitive part word recall score (Kashibayashi et al., 2020). The insula is associated with cognitive functions such as memory and language and closely related to the olfactory response (Menon and Uddin, 2010; Mazzola et al., 2017). In male participants with a normal sense of smell, the cortical thickness of the right insula was related to the discrimination of olfactory quality (Frasnelli et al., 2010). In turn, gray matter loss of insula was found in patients with different forms of olfactory dysfunction, such as that of the left anterior insula in patients with parosmia (Bitter et al., 2011), the right insula in patients with chronic rhinosinusitis and severe olfactory dysfunction (Han et al., 2017), and bilateral insulae in patients with the idiopathic olfactory loss (Yao et al., 2014). Wang and colleagues found that BOLD signals in the primary olfactory cortex (POC), hippocampus, and insula of AD patients were significantly correlated with UPSIT, MMSE, dementia rating scale-2 (DRS-2), and Clinical dementia rating scale (CDR) scores at the lowest odor concentration (Wang et al., 2010). Our study found that the cortical thickness of the left parahippocampal gyrus and bilateral insula were significantly correlated with the CSIT-OI scores and many cognitive test scores.

Olfactory dysfunction is an early predictor of neurodegeneration and is related to late-life cognitive impairment (Doty, 2017). A previous study demonstrated that the declining olfactory activity was correlated with AD structural degeneration in the POC (Vasavada et al., 2015). A more prominent olfactory activity deficit than that of behavioral and tissue volume measurements was shown in the MCI stage. Han et al. (2017) also proved that the olfactory function was significantly correlated with the cognitive level in AD patients (Yoo et al., 2018). In older adults without depression, the impaired olfactory function could predict cognitive decline (Yaffe et al., 2017). Previous work has established that olfactory dysfunction could be used as an early biomarker of Parkinson’s disease (Fullard et al., 2017). Our results were partly consistent with these previous studies that olfactory function was correlated with cognitive functions in overall cognitive level (MoCA), memory (DST), executive control (TMT-A, SDMT), and processing speed (VFT) in T2DM patients. It is speculated that olfactory function in diabetes may influence the cortical thickness of olfaction-related regions involved in cognitive functions, which may be one of the causes of cognitive impairment in T2DM.

This study has several limitations that should be mentioned. First, the course of T2DM patients is variable, which may have an effect on the worsening of cognitive and olfactory performance. Therefore, the course of the disease needs to be more strictly limited in future study. Second, we did not compare the data between T2DM patients with and without olfactory dysfunction. Third, this is a cross-sectional study, which is not enough to prove the causality of this phenomenon. Finally, although we have reported the correlations among cognitive function, olfactory score, and cortical thickness, the biological mechanisms of these correlations are still unclear. Further studies are needed to interpret these results in the future.

## Conclusion

In conclusion, as far as we know, this is the first study using the CSIT method to evaluate the olfactory function in patients with T2DM. We propose that olfactory dysfunction may be useful for future applications that attempt to predict cognitive decline or develop tailored therapies in T2DM patients.

## Data Availability Statement

The original contributions presented in the study are included in the article, further inquiries can be directed to the corresponding author/s.

## Ethics Statement

The studies involving human participants were reviewed and approved by The Research Ethics Committee of the First Affiliated Hospital of Anhui Medical University. The patients/participants provided their written informed consent to participate in this study.

## Author Contributions

MC and JW: methodology, data curation, software, and writing—original draft. SZ, CZ, DD, FL, and WL: data collection, visualization, and investigation. JZ: conceptualization, methodology, software, formal analysis, and writing—review and editing. YY: conceptualization, supervision, and writing—review and editing. All authors contributed to the article and approved the submitted version.

## Conflict of Interest

The authors declare that the research was conducted in the absence of any commercial or financial relationships that could be construed as a potential conflict of interest.

## Publisher’s Note

All claims expressed in this article are solely those of the authors and do not necessarily represent those of their affiliated organizations, or those of the publisher, the editors and the reviewers. Any product that may be evaluated in this article, or claim that may be made by its manufacturer, is not guaranteed or endorsed by the publisher.

## Footnotes

^1^ http://www.neuro.uni-jena.de/cat

^2^ http://www.fil.ion.ucl.ac.uk/spm
